# One Year Outcome and Satisfaction of Presbyopia Correction Using the PresbyMAX® Monocular Ablation Profile

**DOI:** 10.3389/fmed.2020.589275

**Published:** 2020-11-27

**Authors:** Dan Fu, Jing Zhao, Li Zeng, Xingtao Zhou

**Affiliations:** ^1^Eye Institute and Department of Ophthalmology, Eye & ENT Hospital, Fudan University, Shanghai, China; ^2^NHC Key Laboratory of Myopia, Fudan University, Shanghai, China; ^3^Key Laboratory of Myopia, Chinese Academy of Medical Sciences, Shanghai, China; ^4^Shanghai Research Center of Ophthalmology and Optometry, Shanghai, China

**Keywords:** presbyopia correction, monovision, PresbyMAX, refractive outcome, satisfaction

## Abstract

**Purpose:** To explore the safety, efficacy, and satisfaction of the PresbyMAX monocular mode for the correction of presbyopia.

**Methods:** Prospective study. Twenty-two patients (mean age 50.6 ± 6.2 years, 11 myopia patients and 11 hyperopia patients) were enrolled. The dominant eye was fully corrected for distance vision; the non-dominant eye was corrected using central PresbyMAX monocular mode. Binocular uncorrected distance visual acuity (BUDVA), near visual acuity (BUNVA), intermediate visual acuity (BUIVA), corrected distance visual acuity (CDVA), and mean spherical equivalent (SE) were tested at 1 day, 1 week, 1 month, 3 months, and 1 year postoperatively. Questionnaire was performed preoperatively, 1 month, 3 months, and 1 year after surgery.

**Results:** At the final visit, the mean safety index was 1.03 ± 0.14. There were 85.7% eyes with the same or better CDVA than the preoperative value, and 17.1% and 2.9% eyes gained 1 line and 2 lines of CDVA, respectively. All treated eyes achieved 20/25 or better BUDVA, and 95.5% achieved 20/32 or better BUNVA, which improved significantly compared with preoperative values (*P* < 0.001). BUDVA maintained stability from 1 month postoperatively, BUNVA and BUIVA kept stable since 1 week after surgery. Overall satisfaction was 95.5% (21/22) at 3 months visit, and 100% at the last visit. No differences in terms of visual acuity and satisfaction were found between the myopia and hyperopia groups.

**Conclusion:** The PresbyMAX monocular ablation profile was safe and effective in treating presbyopia, with great satisfaction achieved at postoperative 1 year.

## Background

Presbyopia refers to age-related decreased accommodative ability, usually leading to near-vision loss, eye fatigue. Presbyopia severely impacts life quality due to its inevitable and irreversible progression, further posing a financial burden to society (1). It has been estimated that nearly 1.8 billion individuals will suffer from presbyopia by 2050, most of whom live in developing countries (1, 2). In China, the prevalence of presbyopia ranges from 25.2 to 67.3% among individuals > 35 years of age (3, 4). However, presbyopia-correction coverage is relatively low in China, due to the poor quality of available glasses and lack of awareness of presbyopia and its treatment (4).

To date, several surgical methods have been applied to treat presbyopia (5). Monovision and multifocal design are mostly used. Luft et al. (6) firstly reported the safety and efficacy of small incision lenticule extraction (SMILE) monovision. Reinstein et al. (7) successfully applied a hyperopic micro-monovision protocol to treat patients with presbyopia. Other surgeries such as corneal inlay may induce numerous higher-order aberrations, and refractive lens exchange loses accommodation of the crystalline lens. Attempts for producing pseudo-accommodative ability on the cornea induce new correction method of presbyopia by peripheral near zone (concentric ring for near vision) or a central near zone (central disc for near vision) (8, 9).

PresbyMAX (Schwind Eye-Tech-Solutions GmbH and Co., Kleinostheim, Germany) is a technology that combines treatment of presbyopia and ametropia with a bi-aspheric ablation profile using the Schwind AMARIS excimer laser system (10), which has undergone several mode updates. The monocular mode used in the current study is the latest one, known as “PresbyMAX monocular.” However, to the best of our knowledge, few prospective studies have investigated this mode (11). Accordingly, the present prospective study aimed to investigate visual performance (near, intermediate, and far) and satisfaction after PresbyMAX monocular mode treatment.

## Materials and Methods

### Patients

Patients seeking presbyopia correction at EENT Hospital of Fudan University (Shanghai, People's Republic of China) were screened. This prospective and consecutive observational study was approved by review board of EENT Hospital. Informed written consent was obtained from all participants before surgery as a standard protocol. All procedures adhered to the tenets of the declaration of Helsinki. The patients satisfying following criteria were enrolled and followed up throughout to 1 year after surgery.

Inclusion criteria were as follows: age > 40 years, spherical refraction error of −10 to +7 dioptres (D); astigmatism of up to −3 D; stable refraction for 2 years; no use of contact lens within the previous 2 weeks; uncorrected near visual acuity (UNVA) ≤ 20/50, and could increase at least one line with addition power; corrected distance visual acuity (CDVA) ≥ 20/25, strong willing of getting rid of glasses.

The exclusion criteria were the following: intolerance to the preoperative anisometropic test (≥0.75 D), any eye disease except for refraction errors, a history of ocular surgery or trauma.

### Preoperative Assessments

Regular preoperative examinations were performed, including subjective refraction, intraocular pressure, visual acuity, corneal topography, ophthalmoscope, and slit lamp examination.

Visual acuity was tested using a Snellen chart (at 4, 0.8, and 0.33 m, respectively, for distance, intermediate, and near visual acuity) including, corrected distance visual acuity (CDVA), binocular uncorrected distance visual acuity (BUDVA), binocular uncorrected near visual acuity (BUNVA), binocular uncorrected intermediate visual acuity (BUIVA), and binocular distance corrected near visual acuity (BDCNVA). All visual acuities are expressed in logMAR units.

Dominant eye was determined by “hole test” (12). This check is repeated until the results are the same twice in a row. Anisometropia test was conducted by increasing the “add” by 0.25 D interval in the nondominant eyes after distance vision was corrected. Binocular distance and near visual acuity as well as patients' feelings were recorded at each add test.

The surgical eye is based on the patients' subjective feelings during the anismotropia test and individual needs in daily life. The overall design obeys the monovision principle that binocular distance visual acuity is no <20/25 with best near vision as possible. For instance, if there is a patient with SE being +1.0 D in both eyes and having BUDVA of 20/20, then the surgery is only performed on the nondominant eye to improve near vision with no loss of BUDVA.

### Surgical Technique

All surgeries were performed by the same operator (ZXT) between July and November 2017.

The addition was planned and selected within the range of +1.25 to +2.50 D for the nondominant eyes. The surgery consisted two steps: flap creation using Visuamax femtosecond laser (Carl Zeiss Meditec AG, Jena, Germany) and stromal ablation using the Schwind AMARIS 1050RS excimer laser platform with Smart Pulse Technology (Schwind eye-tech-solutions GmbH, Kleinostheim, Germany). The intended flap thickness was 110 μm, with a diameter of 8.0 mm. The patients were then transferred to the Schwind AMARIS platform. A normal aspheric femtosecond-assisted LASIK (FS-LASIK) ablation profile was performed on the dominant eye and a bi-aspheric PresbyMAX ablation profile was performed on the non-dominant eye with the optical zone at 6.2–6.8 mm. After the ablation, the central 3 mm cornea zone of the non-dominant eye was reshaped to a hyper-positive area for near correction, which is influenced by the amount of presbyopia addition. The laser ablation uses a 193-nm flying spot laser system with a super-Gaussian beam profile of 0.54 mm full width at half maximum.

The ablation profile was centered at the corneal vertex using pupillary offset measured with a topographer (Keratron Scout, Optikon, Rome, Italy), which approximates the visual axis. Alignment of the eye with the laser was maintained using an infrared eye tracker with simultaneous limbus, pupil, and torsion tracking integrated into the laser system.

### Postoperative Evaluation

Patients were instructed to wear bandage contact lenses for one night; 0.1% fluorometholone eye drops and artificial tears were applied successively for 3 weeks. Patients were reviewed at 1 day, 1 week, and 1 month, 3 months and 1 year after surgery.

Safety index (postoperative CDVA/preoperative CDVA), which reflects the surgery's safety on visual acuity, was calculated (13).

Satisfaction was subjectively assessed preoperatively, and at 1 month, 3 months and 1 year postoperatively. The questionnaire covered several aspects including: near vision, distance vision, glare; dry eye, halo, and overall satisfaction regarding vision quality (Appendix 1). Each symptom was assessed according to four levels: 0–3, with 0 representing worst and 3 best. Grading criteria refer to the previous study (14).

### Statistical Analysis

Comparisons were performed using the two-tailed Student's *t* test for normal distribution variables, Wilcoxon rank test for abnormal distribution values, and chi-squared or Fisher's exact tests for categorical variables. Repeated univariate analysis of variance was performed to evaluate changes in values at different time-points. The analyses were performed using SPSS software (IBM Corporation, Armonk, NY, USA); *P* < 0.05 was considered to be statistically significant.

## Results

In total, 44 eyes of 22 patients were included, including 11 myopic patients and 11 hyperopic patients. Twenty-two eyes were nondominant eyes that underwent presbymax ablation, and other 13 eyes were dominant eyes targeted to plano underwent regular FS-LASIK. There were nine cases with preoperative refraction of the dominant eyes were plano or within 1.0 D. The nine eyes were unoperated. Basic demographic information of the patients is summarized in Table 1. The mean preoperative spherical equivalent (SE) of the 35 operated eyes was −1.86 ± 4.32 D (−9.88 to 6.88 D), and the mean addition power was 1.56 ± 0.73 D (0.25–2.5 D) (Table 1).

**Table 1:** The basic information of all participants.

**Variables**	
**Age, y**	
Mean (SD)	50.6(6.2)
Range	42–65
**Sex**, ***n*** **(%)**	
Male	6 (27.3)
Female	16 (72.7)
**Spherical diopter, D**	
Mean (SD)	−1.64 (4.31)
Myopia (SD)	−4.93 (2.27)
Hyperopia (SD)	2.73 (1.72)
**Cylinder, D**	
Mean (SD)	−0.66 (0.55)
Myopia (SD)	−0.63 (0.58)
Hyperopia (SD)	−0.72 (0.49)
**Type of surgery**, ***n*** **(%)**	
Bilateral	13 (59.1)
Unilateral	9 (40.9)
**Eye dominance**, ***n*** **(%)**	
Right	15 (68.2)
Left	7 (31.8)
**Ablation mode**, ***n*** **(%)**	
Bi-aspherical eyes	22 (62.9)
Monofocal eyes	13 (59.1)

### Safety

**Figure 1:**
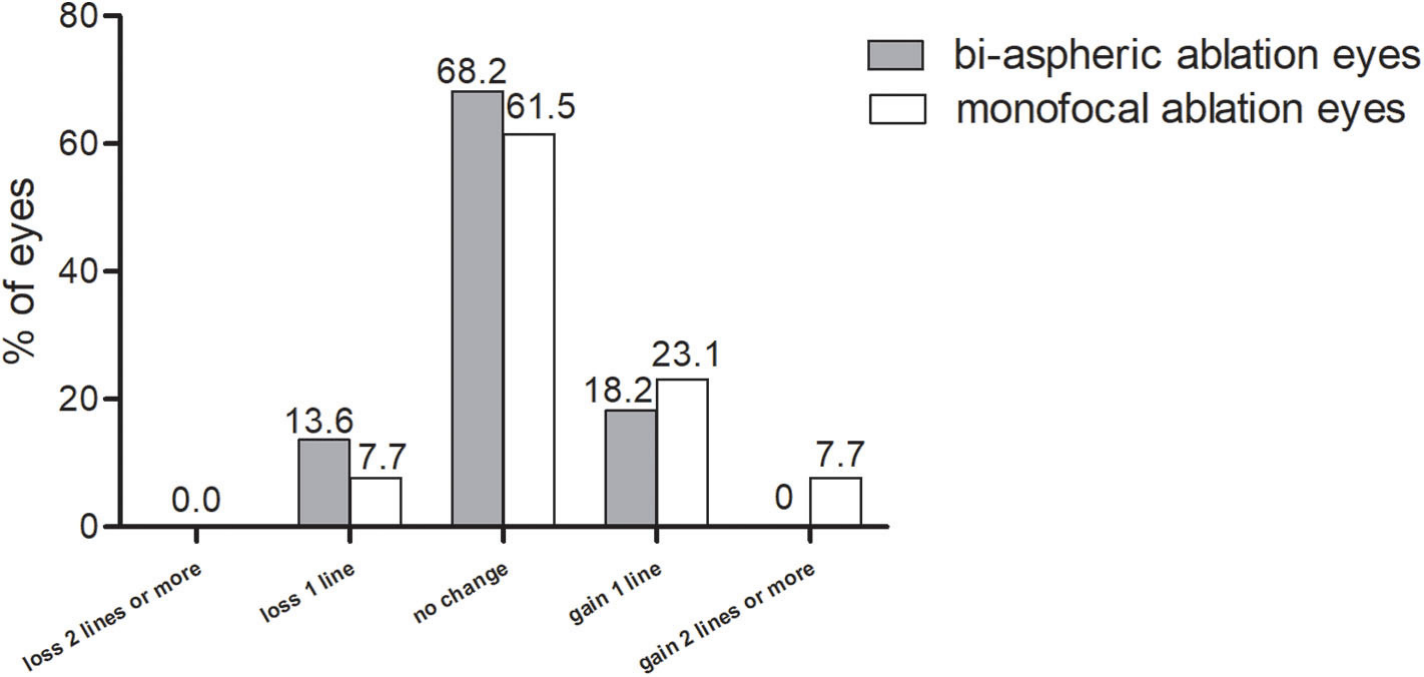
Changes in corrected distance visual acuity at 1 year postoperatively. CDVA, corrected distance visual acuity.

All surgeries were uneventful, without any intraoperative or postoperative adverse events. The CDVA at 1 year postoperatively are shown in Figure 1. The safety index was 1.03 ± 0.14 (range, 0.83–1.5). In total, 65.7% eyes maintained unchanged CDVA, 17.1 and 2.9% eyes gained one and two lines of CDVA, respectively. Among the five eyes losing one line, among which four eyes were bi-aspherical eyes and one eye was monofocal treated. For the five eyes, three myopic eyes and two hyperopic eyes lost one line.

### Efficacy

**Figure 2:**
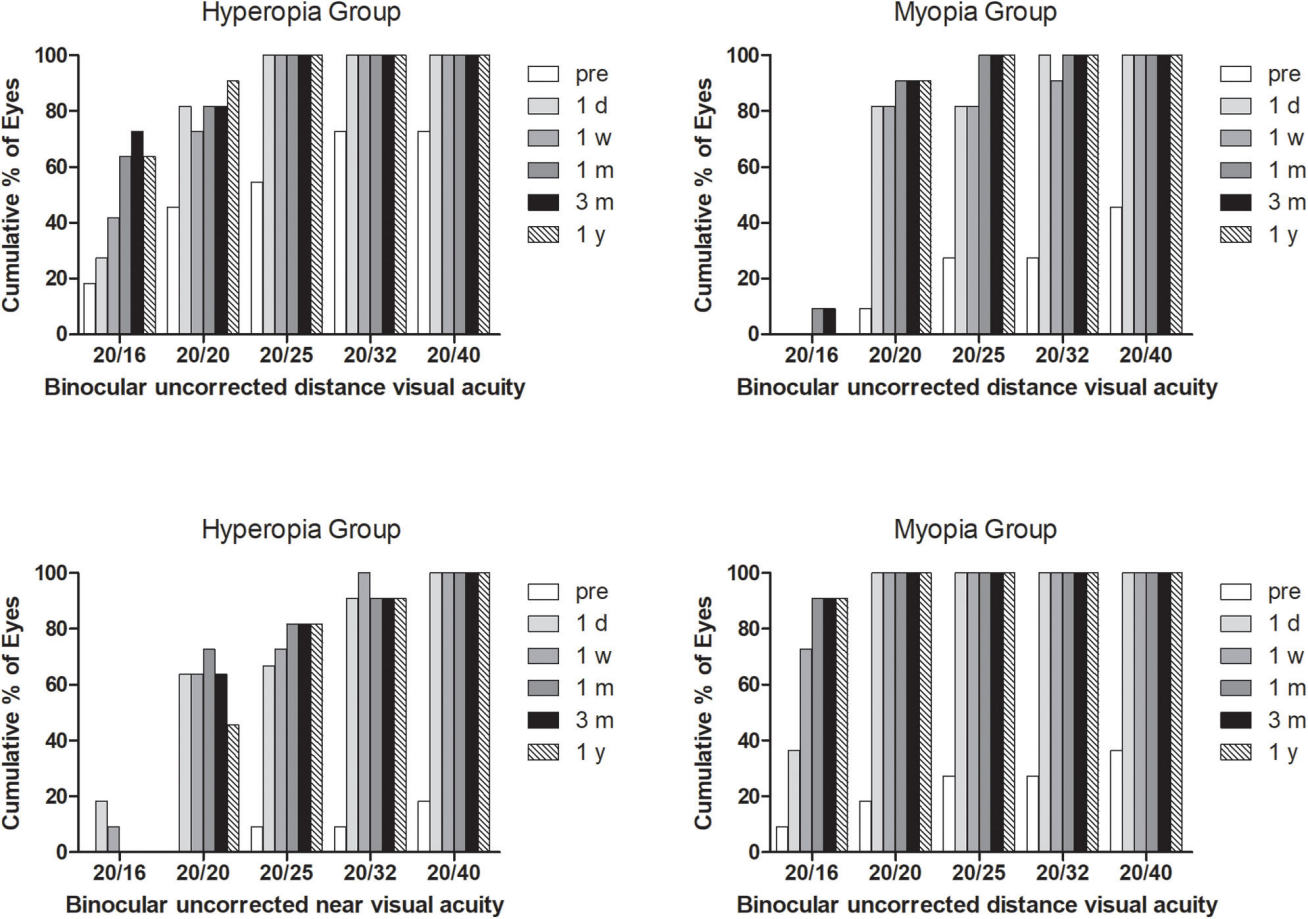
Binocular uncorrected distance and near visual acuity before and after surgery.

At the last visit, BUDVA was equal or better than 20/25 in all patients, and 95.5% patients achieved 20/32 of BUNVA (Figure 2). The BDCNVA at 1 year postoperatively was 0.21 ± 0.18 logMAR, which was worse than the BUNVA (0.05 ± 0.09 logMAR) [*P* = 0.001 (paired *t* test)].

### Stability

**Figure 3:**
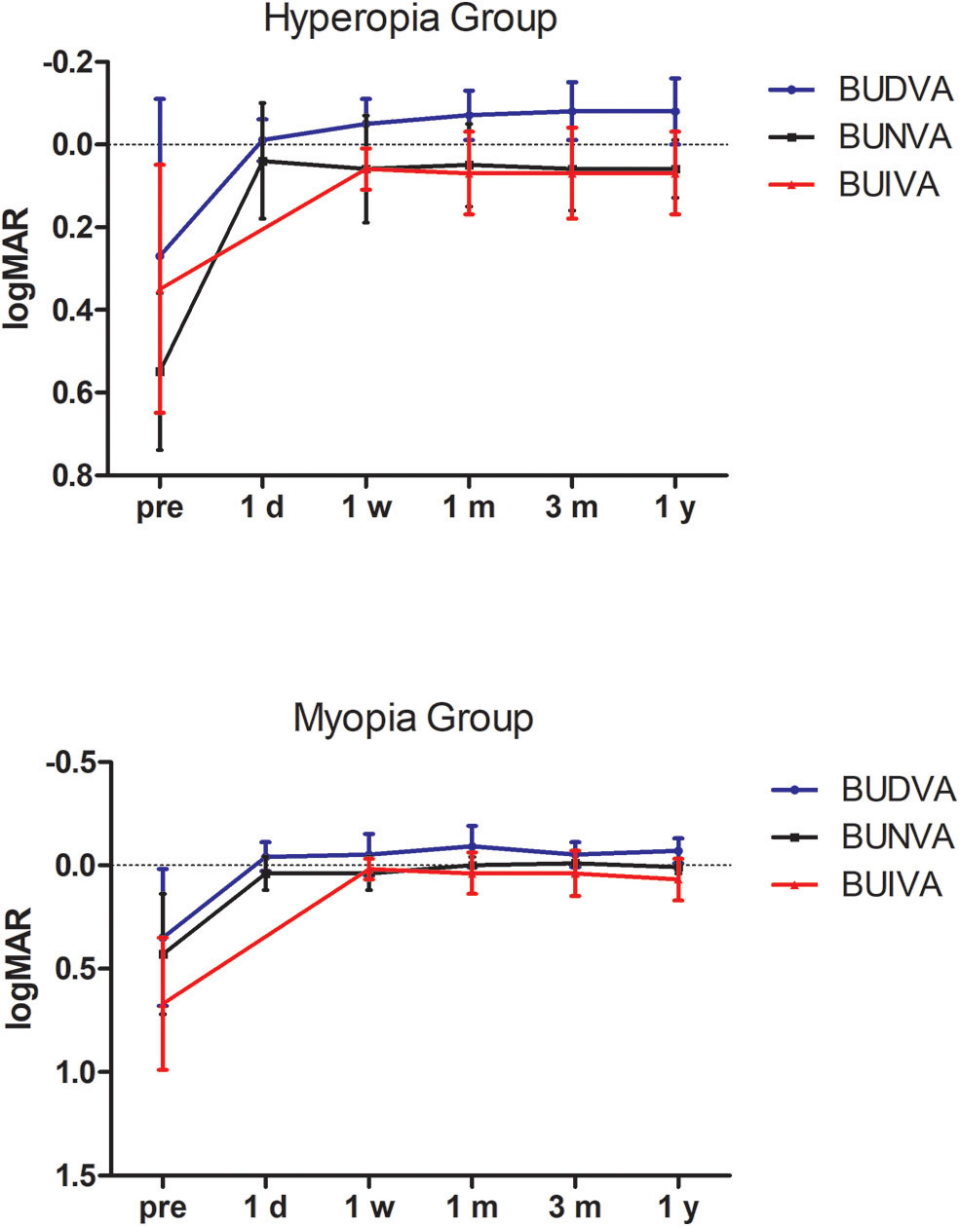
The stability of binocular distance, intermediate, and near visual acuity during follow-up. BUDVA, binocular uncorrected distance visual acuity; BUIVA, binocular uncorrected intermediate visual acuity; BUNVA, binocular uncorrected near visual acuity.

All BUDVA, BUNVA, and BUIVA improved significantly after surgery (*P* < 0.001). There was no significant difference in BUDVA between 1 day and 1 week postoperatively, but BUDVA get improved since 1 month after surgery (post 1 month vs. post 1 day, *P* < 0.01), and kept stable since 1 month after surgery. The BUIVA and BUNVA maintained stable from day 1 after surgery (Figure 3).

### Predictability

**Figure 4:**
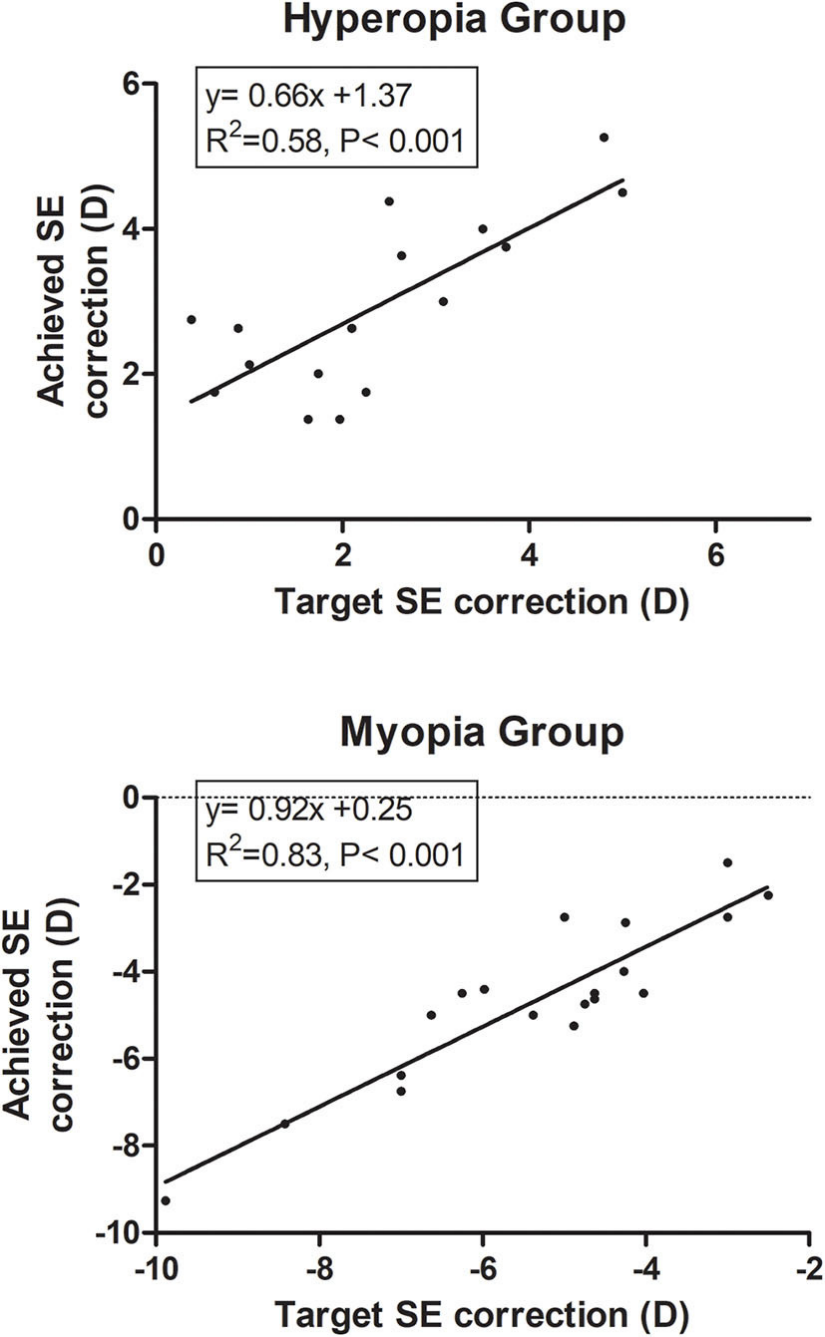
Predictability of spherical equivalent correction.

The target SE correction was strongly correlated with the achieved SE at 1 year after surgery. The linear regression analysis revealed significant predictability (*P* < 0.001). The predictability was better in myopic group (*R*^2^ = 0.83) than hyperopia group (*R*^2^ = 0.58) (Figure 4).

### Accuracy

**Figure 5:**
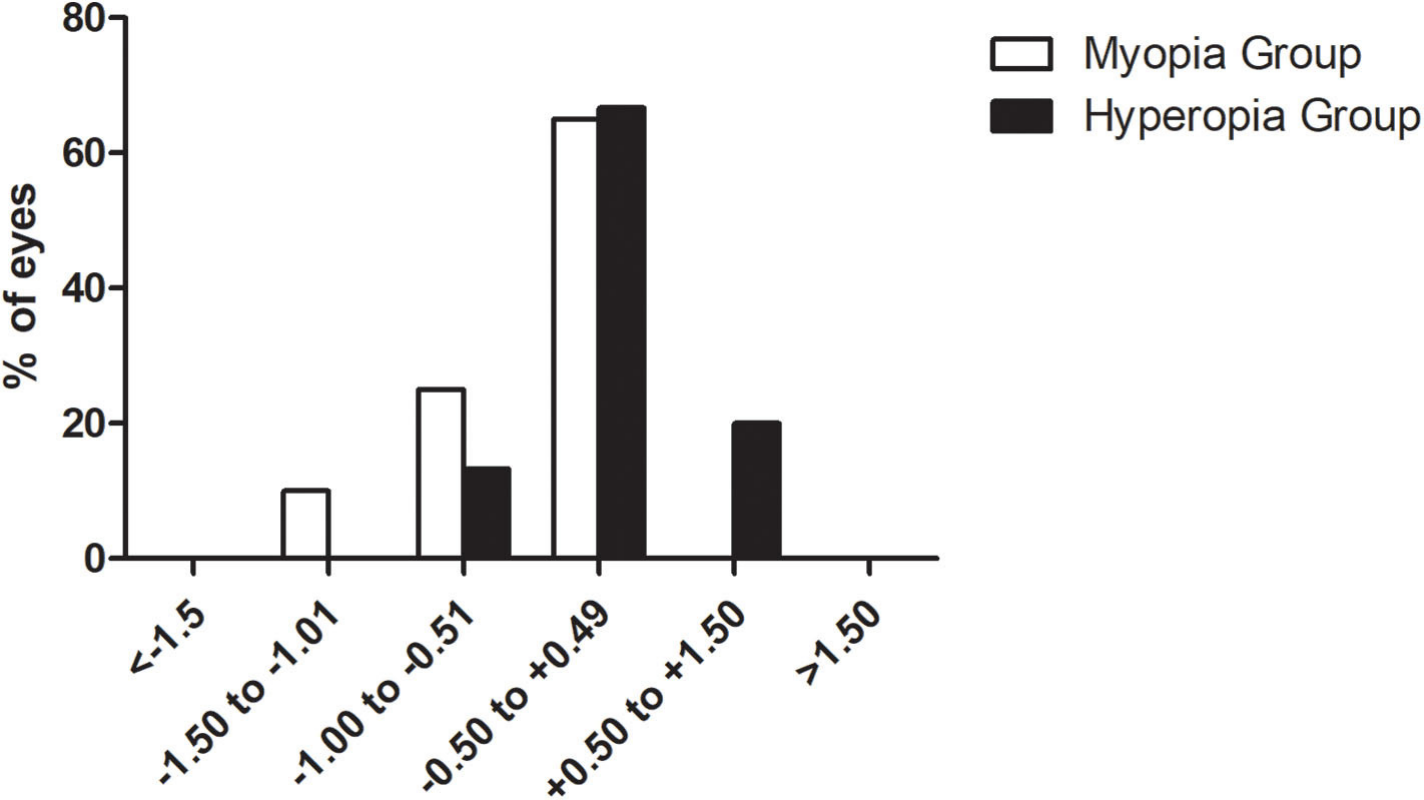
Overall satisfaction regarding vision quality before and after surgery.

The accuracy was shown in Figure 5. Separately, 65% (10/15) myopic eyes and 66.7% (13/20) hyperopic eyes were within ±0.5 D. No eye exceeded the target refraction over 1.5 D.

### Satisfaction Analysis

**Figure 6:**
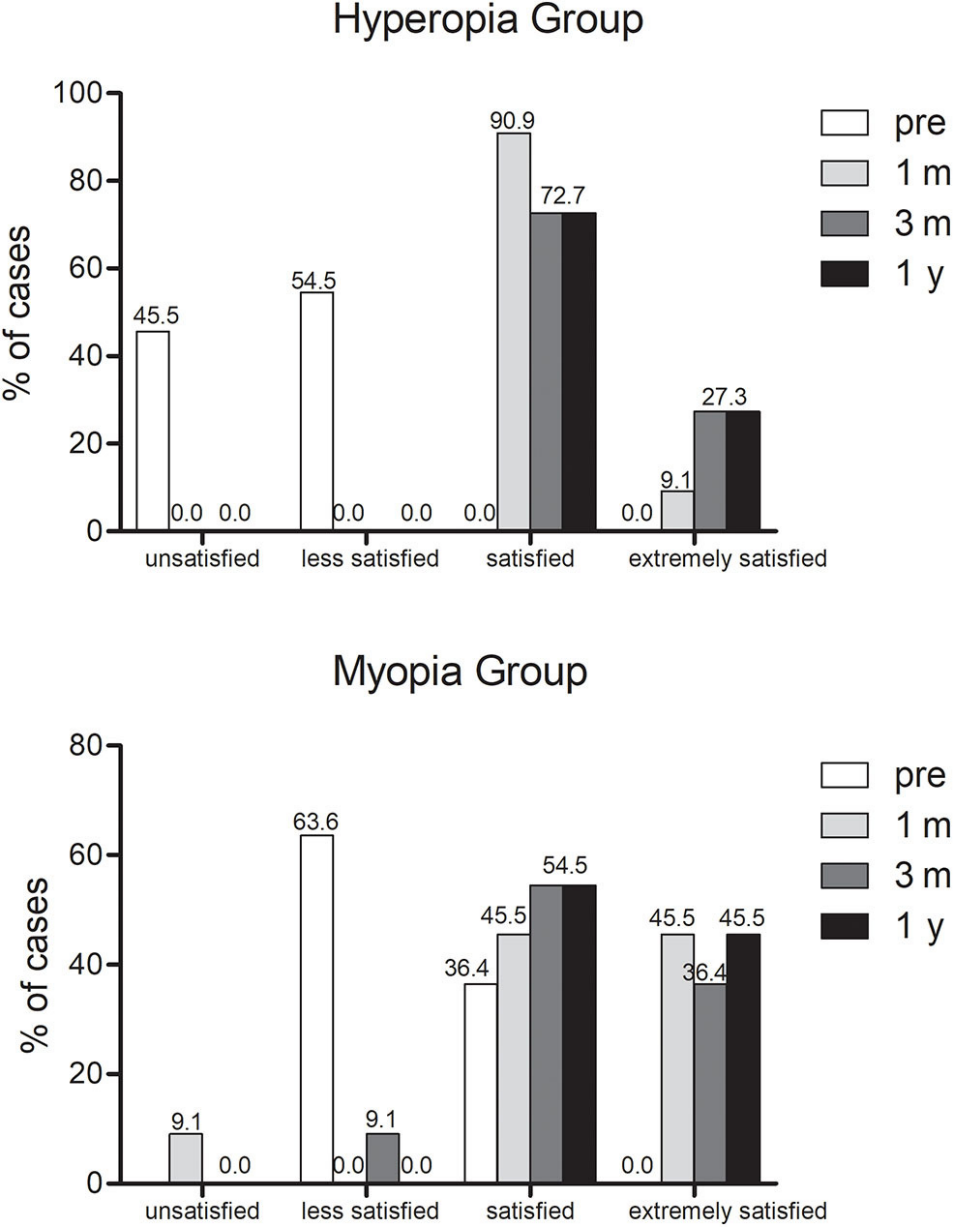
Satisfaction assessment before and after PresbyMAX.

The questionnaire revealed that overall patient satisfaction was 95.5% at 3 months after surgery and 100% at 1 year after surgery (Figure 6). At 3 months visit, only one patient was unsatisfied due to occasional double images, which influenced her daily work. The subjective evaluation for vision quality improved significantly after surgery [*P* < 0.001 (chi-squared test)]. Impaired night vision (6/22), dry eye (6/22), and halo (4/22) were the three most frequent patient complaints at 3 months visit. At postoperative 1 year visit, 18.2% patients (4/22) complained decreased distance vision, 13.6% patients (3/22) complained impaired night vision quality and three other patients suffered from mild dry eye.

### Subgroup Analysis

Patients were further divided into two groups according to preoperative refractive state. There were no differences in visual acuity and satisfaction between the myopia and hyperopia groups (Table 2).

**Table 2:** Comparison between myopia and hyperopia group at 1 year postoperatively.

	**Addition**	**Age**	**BUDVA**	**BUNVA**	**BUIVA**	**Satisfaction**
Myopia (*n* = 11)	1.25 ± 0.8	47.4 ± 4.7	−0.09 ± 0.05	0.01 ± 0.03	0.04 ± 0.12	100%
Hyperopia (*n* = 11)	1.86 ± 0.52	52.5 ± 5.1	−0.06 ± 0.08	0.06 ± 0.07	0.09 ± 0.11	100%
*P*	0.06	0.02	0.30	0.06	0.35	0.66^*^

* *Fisher's exact tests*.

*BUDVA, binocular uncorrected distance visual acuity; BUIVA, binocular uncorrected intermediate visual acuity; BUNVA, binocular uncorrected near visual acuity*.

## Discussion

Presbyopia presents a serious problem for individuals > 40 years of age. Compared with glass correction, refractive surgery has been demonstrated to be an economical and practical method. Nevertheless, there remains a significant challenge for surgeons to optimize the procedure, contributing to better visual acuity and quality of life for individuals with presbyopia. Characterized by comprehensive visual acuity assessment, this study revealed the safety, efficacy, stability, and satisfaction of central PresbyMAX in monocular mode.

The present study yielded a safety index of 1.03 ± 0.14 at postoperative 1 year, and no eyes lost more than two lines of CDVA. Similar results have been reported in previous conventional LASIK procedures. Tomita (15) acquired a short-term safety index of 1.06 among 1,280 eyes treated using the Amaris 750S 750 Hz excimer laser. Lin et al. (16) reported unchanged CDVA 3 months after FS-LASIK. Different laser modes partially explain the minor difference and, as a whole, the safety of LASIK procedures is accepted worldwide. The present study further demonstrated unchanged safety among the presbyopic population. Using the same surgical mode, Chan et al. (11) found no loss of two lines of CDVA after 1 year of surgery in a retrospective investigation, their result was similar to ours.

In this study, all treated eyes achieved 20/25 BUDVA, and 90.9% of eyes achieved 20/25 BUNVA. Visual acuity improved significantly from preoperative levels. Luger et al. (17) found that 1 year after surgery, 93% of cases reached 20/20 or better binocular UDVA; 90 and 97% patients had J2 or better UNVA and UIVA, respectively. Alarcon et al. (18) reported that >90% of patients had a binocular uncorrected distance and near visual acuity of 0.0 logMAR or better 3 months postoperatively. Different from the present study, two studies above applied the Hybrid mode, which intend to induce different increase in depth of focus in both eyes. Decreased contrast sensitivity was the major drawback. When it comes to the previous PresbyMAX symmetry mode (10), the near performance was very good after surgery; however, the distance vision was more affected because of more induced negative spherical aberration and residual myopia target. The PresbyMAX monocular mode in present study can provide rapid vision recovery, and thus, is recommended for populations with higher requirements for distance vision.

Up to 3 months after surgery, the overall satisfaction was 95.5% (21/22) and increased to 100% at 1 year visit. High levels of patient satisfaction have been reported for monovision LASIK (92–96%) (19, 20). Zhang et al. demonstrated comparative satisfaction after crossed and conventional pseudo-phakia monovision (21). Baudu et al. (22) found that ~83% of patients achieved objectively successful outcomes because some patients asked for retreatment to improve distance or near vision. Retreatment also occurred in the study by Chan et al. (11). Most of the retreatment in previous studies occurred at 6 months after surgery. A relative small sample observation may partly explain the 0% retreatment rate in the current study. Baudu et al. (22) revealed that additions of <2.88 D or patients <58 years of age appear to represent the optimum compromise. In this study, no planned addition >2.88 D was used, which may better facilitate adaptation to the new visual impression.

There was one patient who was unsatisfied until 3 months postoperatively for intermittent halo and double images. Although her visual acuity was not bad (BUNVA = 20/20, BUDVA = 20/25), the vision was unable to meet her work needs. Other subjective symptoms, including decreased night vision, dry eye, and halo, in the present study are not unique in PresbyMAX treatment (23). Yoo et al. (24) found impaired night vision in 40% patients after hydrogel inlay. Surgery-induced aberration and decreased contrast sensitivity after surgery, especially in the non-dominant eye were probable mechanisms (18, 25). Distance vision is also influenced by the presence of the central hyper-positive areas when pupil miosis occurs (26). The patient got satisfied at the 1 year visit which reminded us that monovison design probably needs a longer adaption period, and this should be emphasized toward the patient at the preoperative conversation.

By comparing the myopia and hyperopia groups, we found no difference in visual acuity and subjective satisfaction. This is different from the results reported by Baudu et al. in that myopia resulted into better BUNVA, which also correlated with the level of ametropia (22). In contrast, Uthoff et al. (10) found that the hyperopic group was the most satisfied group using objective and subjective tests. By reaching a level close to emmetropia, both BUDVA and BUNVA can improve significantly. For myopic individuals, surgery leads to decreased BUNVA for the correction of myopic refractive error. We did not discriminate the difference possibly because of the small sample size and the different age distribution in the two subgroups. Based on the results, more physiological accommodation is stored in a myopic group as a reason for near visual acuity recovery.

One limitation of the present study is the relatively small sample size. The inclusion criteria were prudent for the tolerance of anisometropia, and only certain individuals are best suited for PresbyMAX. A preoperative trial is necessary to test patient acceptance, and learning about individuals' hobbies and expectations helps to select the proper patient.

## Conclusions

In summary, the PresbyMAX monocular mode was well-tolerated and effective for correcting ametropia with presbyopia. Satisfaction can be achieved postoperatively after a period of adaption. Exploring longer-term results would be valuable.

## Data Availability Statement

The raw data supporting the conclusions of this article will be made available by the authors, without undue reservation.

## Ethics Statement

The studies involving human participants were reviewed and approved by Review Board of EENT Hospital. The patients/participants provided their written informed consent to participate in this study.

## Author Contributions

XZ designed the overall study with contributions from JZ to design the surgical mode. DF carried out examinations and wrote the paper. LZ analyzed the data and revised the manuscript. All authors contributed to the article and approved the submitted version.

## Conflict of Interest

The authors declare that the research was conducted in the absence of any commercial or financial relationships that could be construed as a potential conflict of interest.
