# Supplementary material for: One Year Outcome and Satisfaction of Presbyopia Correction Using the PresbyMAX® Monocular Ablation Profile
Source: Front Med (Lausanne). 2020 Nov 27;7:589275. doi: 10.3389/fmed.2020.589275 (PMC7732614; doi:10.3389/fmed.2020.589275)
Supplement: Supplementary file 1 [file Data_Sheet_1.DOCX]

SMILE-Related Questionnaire

Please choose the following options according to your real feelings in the past week, thank you.
Note: The following items are divided into 4 levels, according to the degree from light to heavy. Grade 0 represents asymptomatic; grade 1 represnets mild and symptomatic, but it does not affect life, and it may not be noticed, but symptoms can be felt after reminding; grade 2 means moderate, symptoms can be felt, moderately affecting normal life, and can be tolerated; grade 3 reprents severe symptoms and a serious impact exists on the quality of life.
If you have any questions, please consult your doctor and choose accurately.

| 1. How clear is your **distance vision** | | | |
| --- | --- | --- | --- |
| 0. perfectly clear | 1. pretty clear | 2. somewhat clear | 3.not clear at all |
| 2. How clear is your **near vision** | | | |
| 0. perfectly clear | 1. pretty clear | 2. somewhat clear | 3.not clear at all |
| 3. How much difficulty do you have **reading small print** on telephone or book | | | |
| 0. no difficulty at all | 1. a little difficulty | 2. moderate difficulty | 3. a lot of difficulty |
| 4. How clear is your **night vision** | | | |
| 0. perfectly clear | 1. pretty clear | 2. somewhat clear | 3. not clear at all |
| 5. How often do the **halos** bothering you that make it difficult to see when you are around bright lights | | | |
| 0. none of the time | 1. a little of the time | 2.most of the time | 3. all the time |
| 6. How often do the **glare** bothering you that make it difficult to see when you are around bright lights | | | |
| 0. none of the time | 1. a little of the time | 2.most of the time | 3. all the time |
| 7. How often do the **blur vision** bothering you that make it difficult to see when you are around bright lights | | | |
| 0. none of the time | 1. a little of the time | 2.most of the time | 3. all the time |
| 8. How often do the **distortion** bothering you that make it difficult to see when you are around bright lights | | | |
| 0. none of the time | 1. a little of the time | 2.most of the time | 3. all the time |
| 9. How often do you experience **dry eye** | | | |
| 0. none of the time | 1. a little of the time | 2.most of the time | 3. all the time |
| 10. How do you like the surgery | | | |
| 0. extremely satisfied | 1. satisfied | 2. a little unsatisfied | 3. unsatisfied at all |

Please rate the surgery (0-10, 10 points is the best)
